# ZNF460-regulated COMMD7 Promotes Acute Myeloid Leukemia Proliferation Via the NF-κB Signaling Pathway

**DOI:** 10.7150/ijms.80047

**Published:** 2023-02-21

**Authors:** Xin Shao, Liang Zhong, Xuan Chu, Peng Wan, Shuyu Chen, Ziwei Zhou, Hongyan Zhang, Meng Wang, Beizhong Liu

**Affiliations:** 1 Central Laboratory of Yongchuan Hospital, Chongqing Medical University, Chongqing 402160, China; 2 Key Laboratory of Laboratory Medical Diagnostics, Ministry of Education, Department of Laboratory Medicine, Chongqing Medical University, Chongqing 400016, China

**Keywords:** Acute myeloid leukemia, COMMD7, ZNF460, NF-κB pathway, Proliferation

## Abstract

Acute myeloid leukemia (AML) is a malignancy of the hematological system, for which there remains an urgent need for new therapeutic and diagnostic targets. COMM domain containing 7 (COMMD7) is a recently-identified oncogene linked to poor prognosis in AML. COMMD7 regulates multiple signaling pathways, including nuclear factor-kappa B (NF-κB) signaling. Here, we report that COMMD7 is highly expressed in the AML cell lines KG1a and U937 and that its inhibition by shRNA reduced proliferation, promoted apoptosis and facilitated cell cycle arrest in the G2/M phase in relation to depression of the NF-κB pathway. Furthermore, zinc finger protein 460 (ZNF460) is overexpressed in AML and regulates COMMD7. We found that knockdown of ZNF460 downregulated the expression of COMMD7 while the NF-κB pathway was also inhibited. In addition, we noticed that knockdown of ZNF460 reduced proliferation and increased apoptosis rate of AML cells and that the cell cycle was blocked in the G2/M phase. In brief, our results revealed a critical effect of the ZNF460-COMMD7-NF-κB axis for the proliferation of AML cells. Therefore, COMMD7 may be a possible therapeutic target for AML.

## Introduction

Acute myeloid leukemia (AML) is a clonally aggressive proliferative disorder of the primitive cells of the hematopoietic lineage, characterized by unlimited proliferation, blocked differentiation and apoptosis 1, 2. AML is the most commonly diagnosed form of acute leukemia in adults 3. Although significant advances in chemotherapy and other drug treatments have been made in recent years, somewhat improving clinical outcomes, AML remains poorly treatable, with a 5-year survival rate of only around 28% owing to high recurrence rates and drug resistance 4-6. It is therefore essential to elucidate the molecular mechanisms by which AML pathogenesis occurs in order to improve existing treatment strategies and to identify new therapeutic targets.

COMM domain-containing protein 7 (COMMD7), a member of the COMMD family, is a highly conserved protein with a copper metabolism gene MURR1 (COMM) domain. The COMMD series of proteins are associated with the regulation of a wide range of biological functions 7, 8. COMMD7 has been previously reported to play a role in a variety of solid tumors 9, 10. It is highly expressed in hepatocellular carcinoma (HCC) where it represses apoptosis and prevents cell cycle arrest by regulating the NF-κB pathway 11, 12. Other studies have found that COMMD7 is overexpressed in pancreatic ductal adenocarcinoma (PDAC) cell lines and correlates with a poor prognosis. Anti-tumor effects can be achieved by suppressing COMMD7 in PDAC cells 13. Recently, bioinformatics analysis has been used to predict that high levels of COMMD7 may be implicated in poor prognosis of AML 14. This implies that targeting COMMD7 may be a potential therapeutic strategy for AML, but its exact mode of expression, biological function and pathogenic mechanisms in AML remain poorly understood. Therefore, the biological function and use of COMMD7 as a potential target for AML therapy deserve further investigation.

Zinc finger proteins (ZNF) form the largest family of transcription factors, with zinc finger structural domains playing an essential part in modulating cell differentiation and embryonic development 15, 16. Recently, increasing reports suggest that ZNF can function as an oncogene in cancer 17-19. In previous studies, excessive expression of ZNF460 was implicated in adverse outcomes of colon cancer and enhanced invasive capacity. ZNF460 transcriptionally upregulates circMTO1 to promote oral squamous cell carcinoma 20. Here, we found that ZNF460 is associated with COMMD7 and regulates its expression, but the exact biological function of ZNF460 is unknown. Therefore, ZNF460 is worth investigating as a potential therapeutic target for AML or as a regulatory molecule of COMMD7.

The current study aimed to explore the impact of COMMD7 and ZNF460 in AML development. It was shown that COMMD7 and ZNF460 are highly expressed in AML cells and promote AML proliferation via activation of the NF-κB pathway.

## Materials and Methods

### Cell culture

Acute myeloid leukemia cell lines NB4, THP1, U937, MV-411and KG1a were from the American Type Culture Collection (ATCC, USA). AML cells were cultured in RPMI-1640 medium (#C11875500BT, Gibco, USA) with 10% fetal bovine serum (#900-108, Gemini, USA) and 1% penicillin-streptomycin (#C0222, Beyotime, China) at 37°C, in 5% CO_2_ 21.

### Cell proliferation assays

The viability of cells was measured with a Cell counting kit-8 (CCK-8) kit (#CA1210, Solarbio, Beijing, China). Infected cells at 5 x 10^3^ per well were seeded into 96-well plates. 10 μL of CCK-8 reagent was added to the wells every 24 h and incubated for a further 2 h protected from light, followed by measurement of optical density (OD) at 450 nm using a microplate reader (BioTeck, CA, USA) 22.

### qRT-PCR

Total RNA from AML cells was isolated using TRIzol reagent (#9108, Takara, Japan), then reverse transcribed into cDNA with PrimeScript RT regent Kits (#RR600, Takara, Japan). RT-qPCR was carried out with SYBR® Premix Ex Taq™ II kits (#RR820A, Takara) in a CFX Connect™ RT-qPCR System (Bio-Rad, USA). All primers were synthesised by Sangon Biotech (Shanghai, China). Primer sequences are given in Table S1. The number of cycles at which the fluorescence intensity increment reaches a threshold in each sample tube was taken as the Ct value. The Ct values of the gene to be tested and the internal reference gene were read separately for each specimen. Using β-actin as the internal reference, the relative expression of the gene to be tested was further calculated for each specimen using 2^-ΔΔCt^ 23.

### Western blotting

Cells were washed thrice using PBS and then lysed for half an hour in a RIPA lysis buffer (#P0013B, Beyotime, China) containing protease inhibitor (#ST505, Beyotime, China) followed by centrifugation at 12,000 rpm for 30 min at 4°C. A BCA kit (#P0010, Beyotime) was then used to determine the protein concentration. Separation of equivalent quantities of total protein samples by SDS-PAGE at a suitable concentration. The protein was subsequently transferred to polyvinylidene difluoride (PVDF) membranes (#BS-PES-45, Millipore, USA). Following blocking for 2 h, appropriate primary antibodies were incubated with the blots for at least 16 h at 4°C. The antibodies employed were as follows: Cleaved-Caspase-3 (#9961,Cell Signaling Technology, USA), PARP (#A5037, Bimake, USA), NF-κB p50/105 (#A5600, Bimake), Bcl2 (#A5010, Bimake), Phospho-CDK1 (#R26267, ZENBIO,China), Caspase3 (#9622, Cell Signaling Technology), NF-κB p65 (#A5075,Bimake), Cleaved-PARP (#A5034, Bimake), Bax (#5023, Cell Signaling Technology), Cyclin B1 (#R23324, ZENBIO, China), MMP9 (#R380831, ZENBIO), ZNF460 (#TD4628, Abmart, China), p38 (#8690, Cell Signaling Technology), COMMD7 (#GTX112076, GeneTex, USA), p21 (#A5163, Bimake), CDK1 (#R23884, ZENBIO), c-Myc (Wanleibio, China), p53 (#A5722, Bimake). GAPDH (#60004-1-Ig, Proteintech, China). The secondary antibody (Biosharp, China) was then incubated with the blot for 1 h at 25℃. Quantification was done using the ECL kit (#WBKLS0500, Millipore, USA) 24.

### Cell infection

AML cells with stable knockdown of COMMD7 and ZNF460 were constructed using recombinant lentivirus (Genechem, Shanghai, China). AML cells were infected with lentivirus and infection enhancer for 48 h. Preliminary determination of infection efficiency by observation of cellular GFP fluorescence, followed by selection of cells with 2 µg/ml of puromycin (#HY-15695, MCE, China) for two weeks was carried out. Follow-up experiments were performed using selected cells. The sequences of shRNA used are listed in Table S2^25^.

### Isolation of peripheral blood mononuclear cells

Peripheral blood was drawn from healthy individuals into anticoagulated blood collection tubes. Peripheral blood mononuclear cells were isolated with human whole blood mononuclear cell separation medium (#LDS1075, TBD, Tianjin, China) 26.

### Flow cytometry

To detect apoptosis, 1×10^6^ AML cells were collected and washed thrice with PBS and resuspended in 1 ml PBS, then stained using Annexin V APC-A and DAPI kit. Apoptosis was detected by flow cytometry (CytoFLEX; Beckman, USA) and data analyzed by CytExpert software (Beckman Coulter, USA) 27.

To assess the cell cycle, 1 × 10^6^ cells were treated with 75% ethanol at 4°C overnight. Following centrifugation, the cell supernatant was discarded and RNase A was added in the dark, incubated at 37°C for 25 min and finally Propidium Iodide (PI, Millipore) was added. Cells were plated in the dark at 4°C for 25 min and the cell cycle was assessed by FACSCaliburTM Flow cytometry (BD Biosciences) 28.

### Co-immunoprecipitation assay

Cells were washed thrice, followed by lysis with IP-Western blot lysis buffer for 30 min. Subsequently cells were centrifugd for 30 min and the supernatant aspirated. Protein A/G Magnetic Beads were washed with PBST thrice and incubated with anti-ZNF460 and anti-IgG (#4414, CST) separately with Protein A/G Magnetic Beads (#16-662, MCE, China) on an inverted mixer for 1 h at 25℃. Beads were washed thrice and subsequently incubated with the supernatant at 4°C for at least 16 h. The next day, proteins were detected by western blotting 29.

### Bioinformatics analysis

UCSC and JASPAR were employed to predict transcription factors that might affect COMMD7. Original data of The Cancer Genome Atlas (TCGA) and Genotype-Tissue Expression (GTEx) were downloaded from UCSC XENA. R 4.1.1 was used to process raw data and subsequent analysis. The GEPIA database was used to analyze the correlation between ZNF460 and COMMD7 30, 31.

### Statistical analysis

All data are from three independent replicate experiments and are presented as mean ± SD. All statistical analyses were performed using unpaired Student's t-tests and one-way ANOVA using GraphPad software (prism8). *p* < 0.05 was regarded as statistically significant (*).

## Results

### COMMD7 is highly expressed and promotes proliferation of AML cells

**Figure 1:**
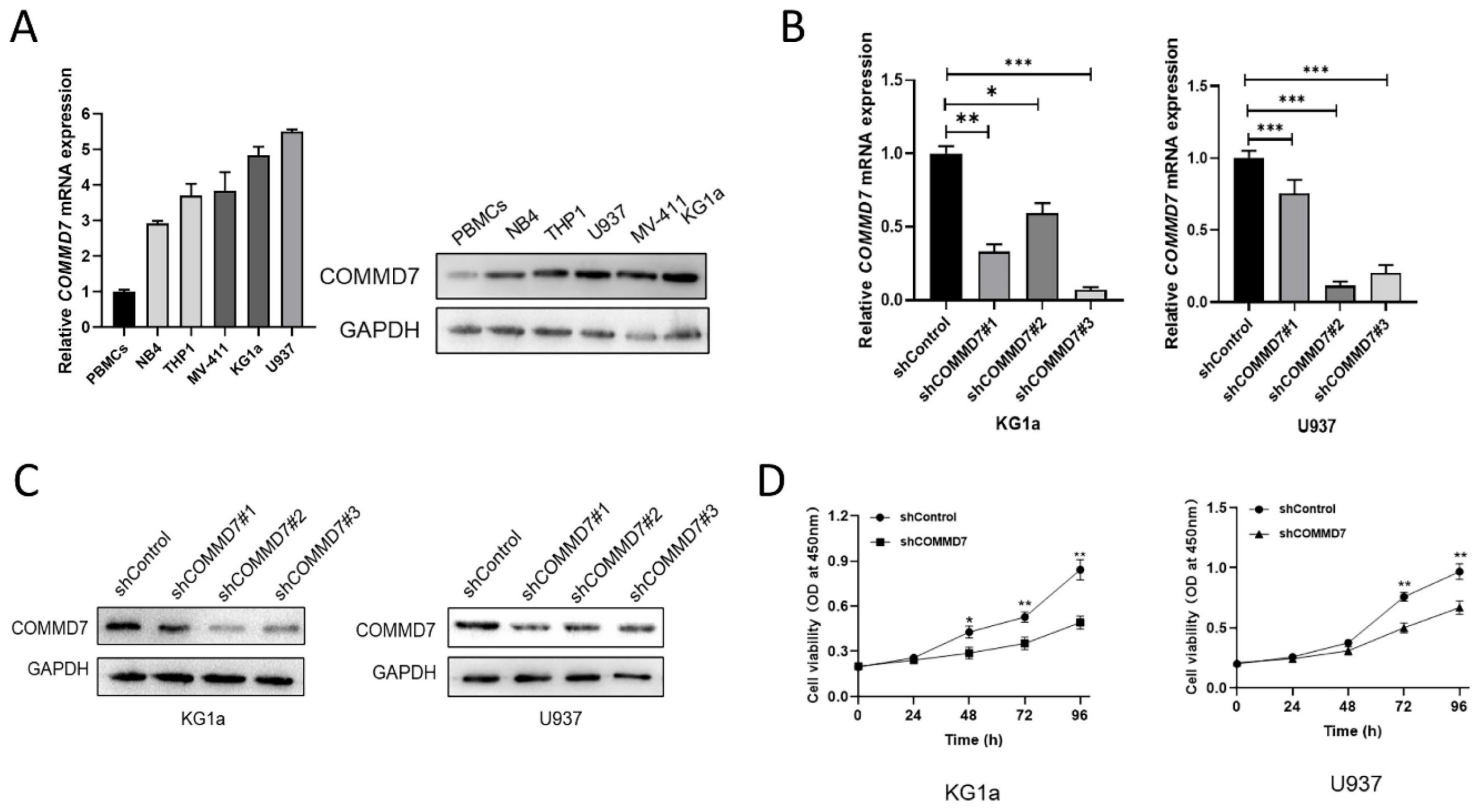
COMMD7 is highly expressed and promotes proliferation of AML cells. The mRNA and protein levels of COMMD7 in NB4, KG1a, THP1, U937, MV-411 and PBMCs (A). Different COMMD7 shRNAs were employed into KG1a and U937 cells. Knock-down efficiency was detected by qRT-PCR (B) and western blotting (C). Knockdown of COMMD7 inhibited KG1a and U937 cells proliferation. Cell viability was analyzed by the CCK-8 assay (D).

It was reported that COMMD7 was predicted to be highly expressed in AML and was implicated in poor survival prospects in previous studies 14. In an effort to explore the relevance of COMMD7 in AML, we first quantified its expression in peripheral blood mononuclear cells (PBMCs) and the AML cell lines NB4, THP1, KG1a, U937 and MV-411. qRT-PCR analysis and western blotting demonstrated markedly higher levels of COMMD7 expression in AML cell lines compared to PBMCs, especially in KG1a and U937 (Fig. 1A). To further study the role of COMMD7 in AML, we knocked it down with lentivirus in U937 and KG1a cells and compared the knockdown efficiency of the three shRNAs at the mRNA and protein levels by qRT-PCR (Fig. 1B) and western blotting (Fig. 1C). We then chose shRNA#3 for the following experiments. Subsequently, we performed CCK-8 assay to examine the impact of COMMD7 on the proliferation of AML cells. The results showed that silencing COMMD7 suppressed the proliferation of KG1a and U937 cells (Fig. 1D). These findings indicate that hyper-expression of COMMD7 facilitates the proliferation of AML cells.

### Knockdown of COMMD7 promotes apoptosis and G2/M arrest via the NF-κB pathway

**Figure 2:**
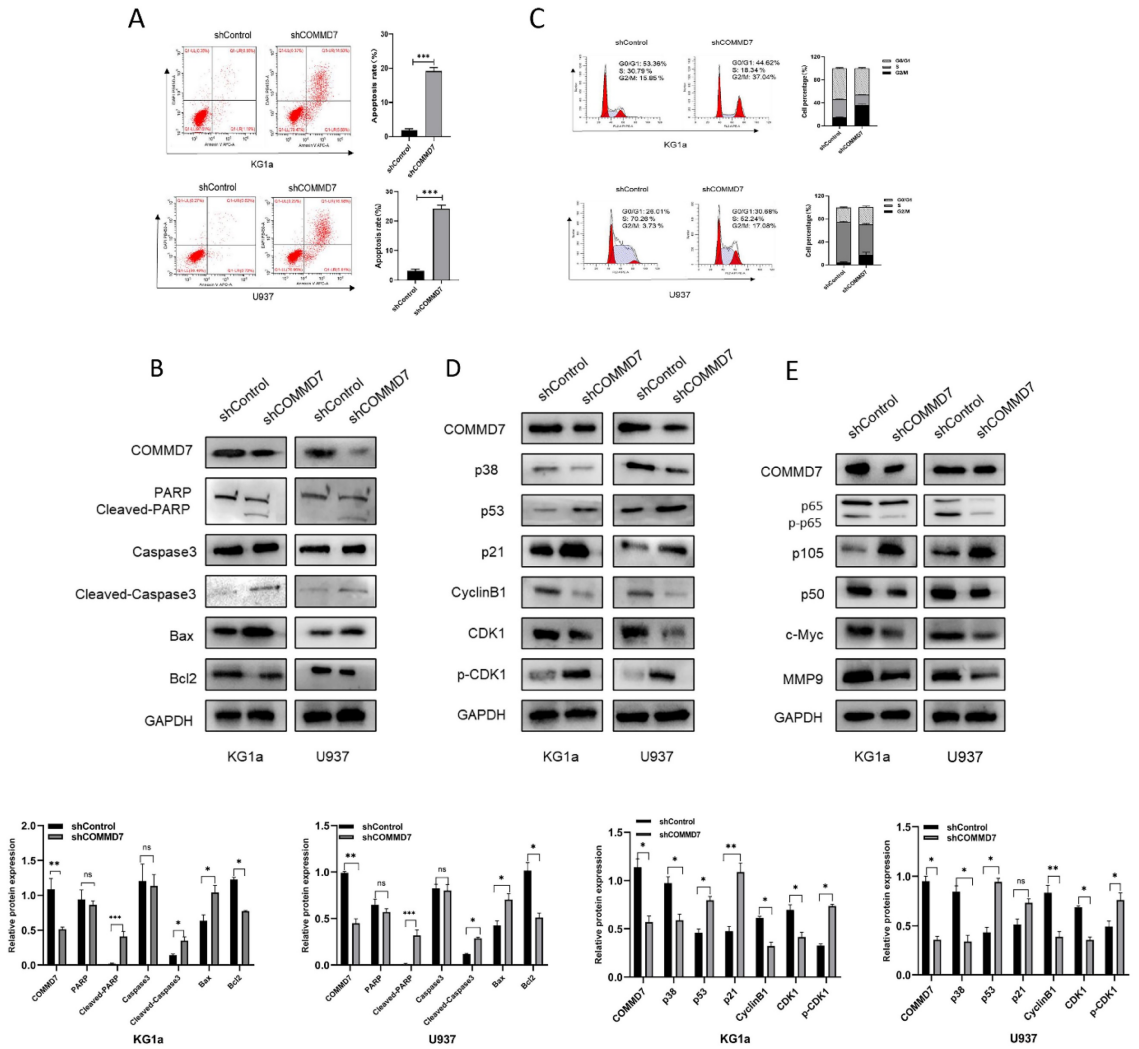
Knockdown of COMMD7 promotes apoptosis and G2/M arrest via the NF-κB pathway. Apoptosis rates of KG1a and U937 were measured by flow cytometry by using annexin V/PI kit (A). Expression of apoptosis-related proteins was tested by western blotting (B). Cell cycle was determined by flow cytometry (C). Western blotting was employed to assayed the cell cycle-associated proteins (D). NF-κB pathway-related protein expression was measured by immunoblotting (E).

Although our results above suggested that knockdown of COMMD7 inhibited proliferation, it was not known exactly how this is achieved. We considered two possibilities: pro-apoptosis and cell cycle arrest. First, AML cells were examined for their apoptosis rate after knockdown of COMMD7. Flow cytometry (FCM) showed that knockdown of COMMD7 led to a significant increase in apoptosis in KG1a and U937 cells (Fig. 2A). Western blotting revealed a marked increase in the expression of Bax, Cleaved-PARP and Cleaved-caspase3, which promote apoptosis, accompanied by a reduction in Bcl2, an apoptosis-suppressing protein (Fig. 2B). Next, the effect of knocking down COMMD7 on the cell cycle was measured by FCM. The outcomes displayed that the proportion of cells in G2/M phase was markedly elevated in both KG1a and U937 cell lines (Fig. 2C). Next, based on the above results, we examined the crucial cyclin-dependent kinases (CDKs) and other molecules that relate to the G2/M phase. Western blotting detected a decrease in Cyclin B1, CDK1 and p38 and an increase in p21, p53 and phosphorylated CKD1 (p-CDK1) (Fig. 2D). This implies that a decrease in CyclinB1/CDK1 activity induces cell cycle arrest in the G2/M phase. This finding is in line with the aforementioned FCM data. Previous work has documented that hyper-expression of COMMD7 activates NF-κB p65 in HCC, so we proceeded to investigate whether the biological function of COMMD7 in AML is achieved through activation of the NF-κB pathway 32. By western blotting, we observed that knockdown of COMMD7 led to a reduction in the phosphorylation level of p65 and the expression of NF-κB p50. An increase in p105, a precursor of p50, was also detected. Some additional proteins were reduced to some extent as well, such as c-Myc and MMP9 (Fig. 2E). In conclusion, knockdown of COMMD7 in AML cells inhibits proliferation, promotes apoptosis and blocks the cell cycle, an effect likely achieved by the suppression of the activation of the NF-κB pathway.

### ZNF460 interacts with COMMD7 and promotes AML cell proliferation

**Figure 3:**
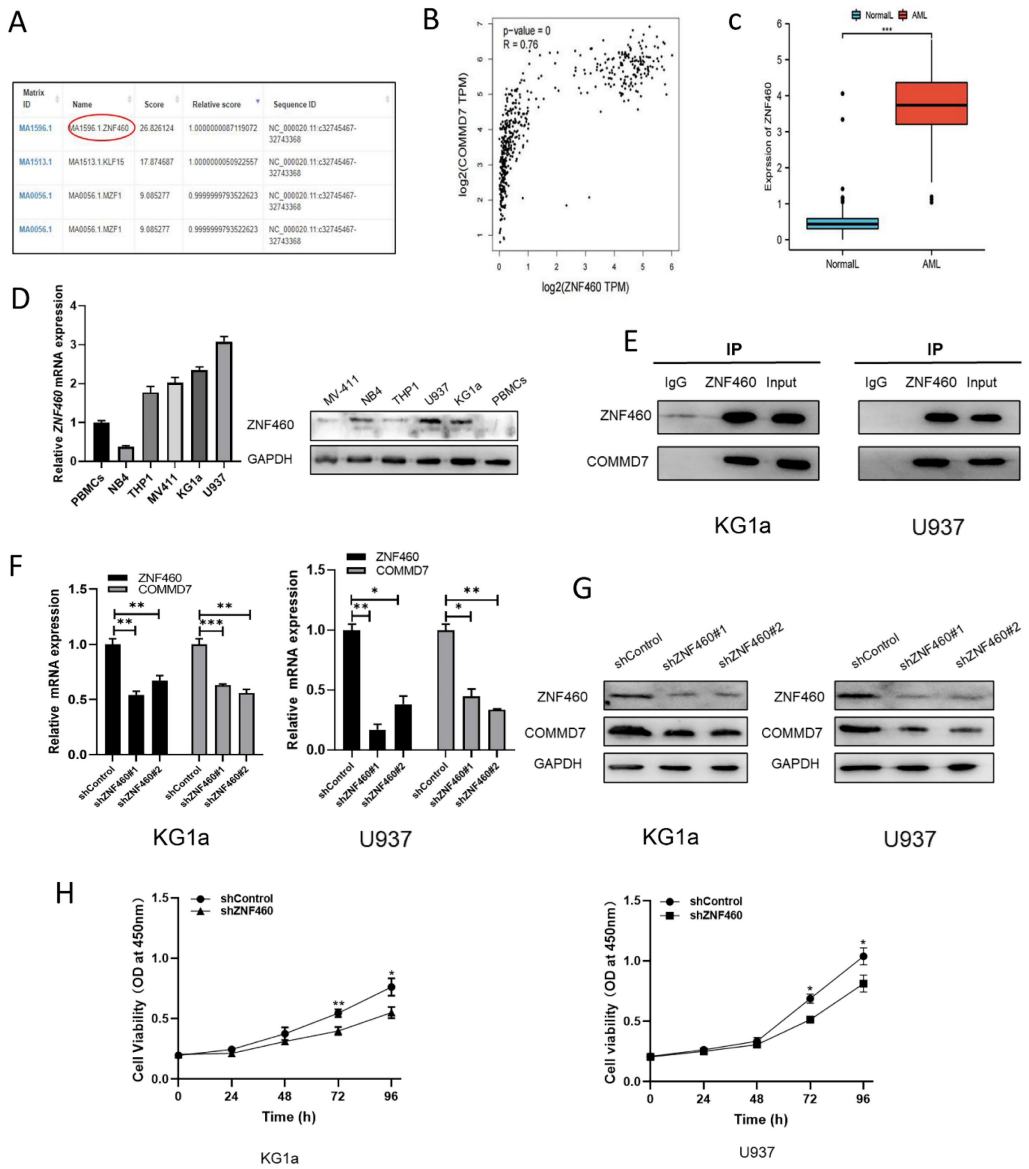
ZNF460 interacts with COMMD7 and promotes AML cell proliferation. Potential transcription factors of COMMD7 promoter were projected through linking UCSC database with JASPAR website (A). Correlation between ZNF460 and COMMD7 was analyzed by GEPIA (B). Expression of ZNF460 in normal and AML groups (C). Expression of ZNF460 in AML cell lines was assessed by qRT-PCR and western blotting (D). Protein extracted from KG1a and U937 cells was co-immunoprecipitated with anti-ZNF460 antibody and blotted with anti-ZNF460 and anti-COMMD7 antibodies (E). Knockdown efficiency of ZNF460 in KG1a and U937 cells and expression of COMMD7 after knockdown of ZNF460 were detected by qRT-PCR (F) and western blotting (G). CCK-8 assays were used to assess cell proliferation (H).

We next explored the upstream regulatory mechanisms of COMMD7 action in AML cells. By means of the bioinformatics tools UCSC and JASPAR we identified transcription factor ZNF460 as upstream of COMMD7 (Fig. 3A). We then analyzed the correlation between ZNF460 and COMMD7 expression using the bioinformatics tool GEPIA and found that they were highly correlated (Fig. 3B). Next, we analyzed the expression of ZNF460 by normal samples in the TCGA and GTEx databases and corresponding AML samples in the former and found that it was highly expressed in AML (Fig. 3C). The qRT-PCR and western blotting results confirmed these predictions (Fig. 3D). Interactions between ZNF460 and COMMD7 were next investigated using co-immunoprecipitation (Co-IP) assays. Immunoprecipitation with anti-ZNF460 antibody pulled down endogenous COMMD7 in both KG1a and U937 (Fig. 3E). To investigate the biological function of ZNF460, we knocked it down in these cell lines. We found that using shZNF460#1, with the best knock-down efficiency, resulted in downregulation not only of ZNF460 but also COMMD7, at both the mRNA and protein levels (Fig. 3F-G). Next, we used the CCK-8 assay to detect the effect of knocking down ZNF460 on the proliferation of KG1a and U937 cells and found that their growth was significantly inhibited. Taken together, these data suggest that the highly expressed ZNF460 upregulates COMMD7 and promotes AML cell proliferation.

### The effect of ZNF460 downregulation on apoptosis and cell cycle in AML cells is mediated via the NF-κB pathway

**Figure 4:**
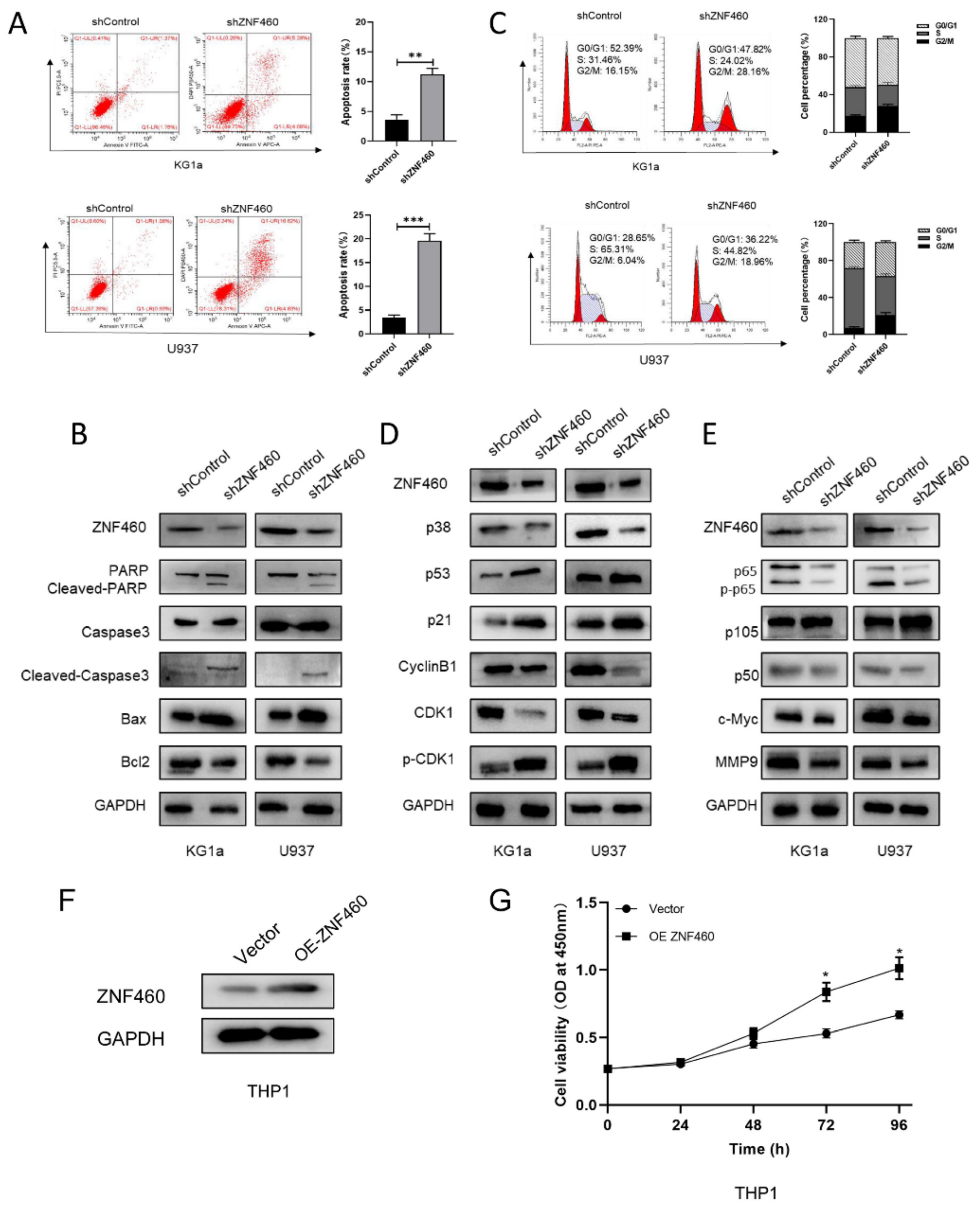
The effect of ZNF460 downregulation on apoptosis and cell cycle in AML cells is mediated via the NF-κB pathway. Apoptosis rates of KG1a and U937 were measured by flow cytometry by using annexin V/PI kit (A). Expression of apoptosis-related proteins was tested by western blotting (B). Cell cycle was determined by flow cytometry (C). Western blotting was employed to assayed the cell cycle-associated proteins (D). NF-κB pathway-related protein expression was measured by immunoblotting (E). Western blotting was used to detect overexpression of ZNF460 (F). CCK-8 assay was used to assess cell proliferation (G).

To explore the manner by which knocking down ZNF460 inhibits proliferation, we assayed apoptosis and the cell cycle as before. The outcomes suggested that the silencing of ZNF460 promoted apoptosis in AML cells (Fig. 4A). Western blotting documented that knockdown of ZNF460 resulted in an increase in Cleaved-caspase3, Bax and Cleaved-PARP, along with a corresponding decrease in some apoptosis-inhibiting proteins, such as Bcl2 (Fig. 4B). Cell cycle assays using FCM showed that knockdown of ZNF460 also leads to cell cycle blockade at the G2/M phase (Fig. 4C). Therefore, we monitored the expression of the relevant proteins by western blotting. The findings suggested that there was a reduction in the expression of Cyclin B1 and CDK1 and an increase in p-CDK1, implying a decrease in CDK1/Cyclin B1 activity. Corresponding changes were seen for other molecules, such as p53 and p21, which inhibit the Cyclin B1/CDK1 complex (Fig. 4D). Finally, we explored the mechanisms by which ZNF460 plays a role in AML and, given the above results, we examined the regulation of the NF-κB pathway by knocking down ZNF460. The findings also suggested a decrease in p65 and phosphorylated p65 (p-p65), implying that both the expression and the activity of p65 were suppressed. A decrease in p50 and an increase in its precursor p105 could also be detected. c-Myc and MMP9 were all downregulated to some extent (Fig. 4E). In a further study of the definitive role of ZNF460 in AML proliferation, we overexpressed ZNF460 in THP1 cells, a leukemic cell line with low expression of ZNF460. Western blotting showed successful overexpression of ZNF460 (Fig. 4F). Finally, we analyzed the effect of overexpression of ZNF460 on proliferation using CCK-8 assay and found that the proliferation of THP1 cells was promoted to some extent following the overexpression of ZNF460 (Fig. 4G). In conclusion, knockdown of ZNF460 inhibits AML cell proliferation by promoting apoptosis and cell cycle arrest.

## Discussion

AML is the most common form of acute leukemia in adults, caused by radiation, genetic mutations or chemicals 33. Treatment with chemotherapy, standard intensive 7+3 therapy, stem cell transplantation and targeted drugs is commonly used, but there are many cases of poor patient prognosis with low five-year survival rates and drug resistance 34, 35. In addition, the heavy burden on patients and the severe side effects of treatment are also serious problems 36, 37. The molecular mechanisms by which AML develops are not thoroughly understood, and there is still a lack of effective targets for AML treatment 38, 39. Therefore, finding a promising therapeutic target is a pressing issue. In this report, the involvement of ZNF460 and COMMD7 in AML was investigated for the first time. The findings confirmed the hypothesis that the ZNF460-COMMD7-NF-κB axis affects AML cell proliferation and provide a new approach for the modulation of the NF-κB signaling pathway.

Mounting evidence points to an influential role for COMMD7 in promoting tumor progression 40. In a recent report, it was shown that knocking down COMMD7 in HCC stem cells inhibited their proliferation, migration and invasion* in vitro*, and also tumor progression *in vivo* 32. Recently, Cao and colleagues found that abnormal expression of COMMD7 has an important role in gastric cancer. Either targeting miR-514a-5p by Linc00852 to regulate COMMD7 expression or directly knocking out COMMD7 heightened the sensitivity of GC cells to DPP, thus facilitating GC therapy 41. In the present study, we characterized COMMD7 as an emerging regulatory molecule for the development of AML. Our results documented that after knockdown of COMMD7, proliferation of KG1a and U937 cells was inhibited, apoptosis was elevated, and the cell cycle was arrested in the G2/M phase. In line with previous studies, COMMD7 has an activatory effect on the NF-κB pathway, but the mechanism by which COMMD7 functions in AML is not clear. In their study, Hawe et al. noted that COMMD7 can be disturbed by genetic sequence variants and abnormal DNA methylation, which can affect white blood cell counts. This is consistent with the abnormal white cell count in AML and may suggest a promising potential mechanism involving COMMD7 in AML 42. This deserves further investigation. Taken together, our findings suggest that COMMD7 facilitates AML development.

A growing number of studies are identifying ZNFs as proto-oncogenes for many different tumors, associated with poor disease prognosis. Previous studies have shown that certain ZNFs have multiple effects in promoting tumor proliferation, migration and invasion. For example, LINC00857 regulated by ZNF460 promotes migration, invasion and epithelial mesenchymal transition of pancreatic cancer cells by upregulating CLDN12 expression through sponging miR-150-5p and recruiting SRSF1 43. Hao and co-workers have shown that hyperexpression of ZNF460 is related to adverse prognosis and that it facilitates cell migration in colon cancer via the JAK2/STAT3 signaling pathway 44. Nevertheless, the exact levels of expression and molecular functionality of ZNF460 in AML remain unclear. Here, we report for the first time that ZNF460 is overexpressed in AML cell lines, and that it mobilizes the NF-κB pathway. In addition, we found that ZNF460 functions to boost AML by upregulating COMMD7. However, the exact mechanism involved is still not clear. Nonetheless, the above findings demonstrate that ZNF460 is a promising biomarker and therapeutic target in AML.

Taken together, our study found that COMMD7 and ZNF460 were overexpressed in AML cells and that this was associated with their proliferation. However, due to limited conditions, we did not detect the expression of ZNF460 or COMMD7 in primary cells *ex vivo* in our study and will refine it in the future. They have a role in boosting growth and progression through the cell cycle, in inhibiting apoptosis and activating the NF-κB pathway. In previous studies, COMMD7 was considered to be a regulatory protein of the NF-κB pathway, but the mechanisms involved were not clarified 8, 45, 46. We have attempted to investigate this issue and have used bioinformatics to screen for ZNF460, a transcription factor that regulates COMMD7, showing that ZNF460 upregulates the mRNA and protein expression of COMMD7. Taken together, these data suggest that ZNF460 and COMMD7 may be therapeutic targets for AML, and we will proceed with in-depth studies on the specific regulatory mechanisms of ZNF460 on COMMD7 and abnormal DNA methylation.

## Conclusions

In conclusion, we found that aberrant high expression of COMMD7 promotes AML cell proliferation and cell cycle progression, inhibits apoptosis and activates the NF-κB signaling pathway. ZNF460 up-regulates COMMD7 to promote AML development.

## Supplementary Material

Supplementary tables.

## Abbreviations

AML: acute myeloid leukemia
COMMD7: COMM domain containing 7
Co-IP: co-immunoprecipitation
PVDF: polyvinylidene difluoride
qRT-PCR: quantitative real-time PCR
ZNF460: Zinc finger proteins 460
HCC: hepatocellular carcinoma
NF-κB: nuclear factor-kappa B
PDAC: pancreatic ductal adenocarcinoma
PBS: Phosphate Buffered Saline
CDKs: cyclin-dependent kinase
CCK-8: Cell counting kit-8
GTEx: Genotype-Tissue Expression
TCGA: Cancer Genome Atlas
PBMCs: peripheral blood mononuclear cells
p-CDK1: phosphorylated CKD1
p-p65: phosphorylated p65
OD: optical density
FCM: Flow cytometry
