# Supplementary material for: ZNF460-regulated COMMD7 Promotes Acute Myeloid Leukemia Proliferation Via the NF-κB Signaling Pathway
Source: Int J Med Sci. 2023 Feb 21;20(4):520–9. doi: 10.7150/ijms.80047 (PMC10087627; doi:10.7150/ijms.80047)
Supplement: Supplementary file 1 — Supplementary tables. [file ijmsv20p0520s1.pdf]

Table S1 The primer sequences used in this experiment are as follows.

Genes	Sequences (5'-3')
COMMD7	F:5'-TCCAGGCGGATTTCATAACTCT-3'
	R:5'-CACTTTCTCCAATTCGCTGCT-3'
ZNF460	F:5'- CTCATTCGACACTTCAACATCC-3'
	R:5'-GTGGATGCTAAAGTGTCGAATC-3'
β-actin	F:5'-CTCCATCCTGGCCTCGCTGT-3'
	R:5'- GCTGTCACCTTCACCGTTCC-3'

Abbreviations: F stands for forward; R stands for reverse

Table S2 The shRNA sequences used in this study are as follows.

Genes	Sequence
COMMD7#1	5'-GTTGGTGGTTAAGAAAGGAAA-3'
COMMD7#2	5'-CAGAACCAGCATGGAGTGTTT-3'
COMMD7#3	5'-GCTCATAGATATGGAGTGGAA-3'
ZNF460#1	5'-GCGACAGCTGATGGTATTTGT-3'
ZNF460#2	5'-GCATAACAAGAGCCACAATGA-3'
